# Enhancement in the Efficiency of Sb_2_Se_3_ Solar Cells by Triple Function of Lithium Hydroxide Modified at the Back Contact Interface

**DOI:** 10.1002/advs.202304246

**Published:** 2023-09-10

**Authors:** Huafei Guo, Shan Huang, Honcheng Zhu, Tingyu Zhang, Kangjun Geng, Sai Jiang, Ding Gu, Jian Su, Xiaolong Lu, Han Zhang, Shuai Zhang, Jianhua Qiu, Ningyi Yuan, Jianning Ding

**Affiliations:** ^1^ School of Microelectronics and Control Engineering Jiangsu Collaborative Innovation Center for Photovoltaic Science and Engineering Jiangsu Province Cultivation base for State Key Laboratory of Photovoltaic Science and Technology Changzhou University Changzhou 213164 China

**Keywords:** carrier collection, deep‐level defect, gradient band structure, Li gradient field, Sb_2_Se_3_

## Abstract

The efficiency of antimony selenide (Sb_2_Se_3_) solar cells is still limited by significant interface and deep‐level defects, in addition to carrier recombination at the back contact surface. This paper investigates the use of lithium (Li) ions as dopant for Sb_2_Se_3_ films, using lithium hydroxide (LiOH) as a dopant medium. Surprisingly, the LiOH solution not only reacts at the back surface of the Sb_2_Se_3_ film but also penetrate inside the film along the (Sb_4_Se_6_)_n_ molecular chain. First, the Li ions modify the grain boundary's carrier type and create an electric field between p‐type grain interiors and n‐type grain boundary. Second, a gradient band structure is formed along the vertical direction with the diffusion of Li ions. Third, carrier collection and transport are improved at the surface between Sb_2_Se_3_ and the Au layer due to the reaction between the film and alkaline solution. Additionally, the diffusion of Li ions increases the crystallinity, orientation, surface evenness, and optical electricity. Ultimately, the efficiency of Sb_2_Se_3_ solar cells is improved to 7.57% due to the enhanced carrier extraction, transport, and collection, as well as the reduction of carrier recombination and deep defect density. This efficiency is also a record for CdS/Sb_2_Se_3_ solar cells fabricated by rapid thermal evaporation.

## Graphical abstract

Surprisingly, the lithium hydroxide solution, which is also used for corroding the Sb_2_Se_3_ film, can increase the efficiency (the highest efficiency of an Sb_2_Se_3_ solar cell based on the RTE method) of the device by optimizing the back contact, decreasing deep‐level defects, and forming a gradient band structure.

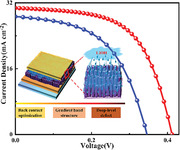

## 1 Introduction

Antimony selenide (Sb_2_Se_3_) thin films are a promising choice for low‐cost, high‐efficiency thin‐film solar cells. Other desirable optoelectronic characteristics of Sb_2_Se_3_ film include a sufficient bandgap (1.03 eV) and a high absorption coefficient (>10^5^ cm^−1^). These materials are made up of plentiful, non‐toxic, and inexpensive component elements.^[^ ^1^, ^2^, ^3^, ^4^, ^5^, ^6^ ^]^ In recent years, there has been significant progress in increasing the efficiency of Sb_2_Se_3_ thin‐film solar cells.^[7]^ The highest efficiency reported so far for Sb_2_Se_3_ solar cells is 10.57% for an superstrate structure, in which the Sb_2_Se_3_ film was prepared using the chemical bath deposition (CBD),^[8]^ and 10.15% for a substrate structure, in which the Sb_2_Se_3_ film was prepared using the injection vapor deposition (IVD) method.^[9]^ However, these efficiencies are still far below the theoretical limit of 32.74% for Sb_2_Se_3_ solar cells.^[10]^ Therefore, increasing efficiency remains the most critical aspect for the further development of this devices.^[11]^

The efficiency of Sb_2_Se_3_ solar cells is restricted by several factors. One of the primary causes is the high deep‐level defect density of the Sb_2_Se_3_ film, which results in non‐radiative carrier recombination, reducing the solar cell's efficiency.^[^ ^12^, ^13^ ^]^ Another reason is the high carrier recombination at the device's interface, leading to decreased carrier transport and collection at the heterojunction interface and back surface.^[14]^ Numerous studies have addressed these two issues, such as the in situ passivation to decrease deep‐level defect density by IVD and CBD methods.^[^ ^8^, ^9^ ^]^ However, these methods are unsuitable for large‐scale and low‐cost industrial production. Therefore, developing a simple and cost‐effective deep‐level defect passivation method is critical for increasing the efficiency and further development of Sb_2_Se_3_ solar cells. Moreover, doping in semiconductors is essential for band energy engineering to address the issue of device interface defect passivation. Several elements have been used as dopants in Sb_2_Se_3_ and cadmium sulfide (CdS) films.^[^ ^15^, ^16^, ^17^ ^]^ However, the fermi level pinning effect caused by deep‐level defects in Sb_2_Se_3_ film hinders effective band structure adjustment through doping, leading to insignificant improvement in the efficiency of Sb_2_Se_3_ solar cells. Therefore, effective methods to modify the band alignment at the Sb_2_Se_3_/CdS interface are urgently needed to increase the open circuit voltage (*V*_OC_) of Sb_2_Se_3_ solar cells further.^[18]^ Furthermore, carrier collection at the back surface is still limited by the back contact barrier between the Sb_2_Se_3_ film and high work function Au electrode, thus, restricting the efficiency increase of Sb_2_Se_3_ solar cells.^[19]^ Constructing the p‐i‐n structure by selecting an appropriate hole transport layer to form a ladder band arrangement can significantly improve the device's charge carrier collection. The organic material Spiro‐OMeTAD acts as the hole transport layer in the record efficiency of Sb_2_S_3_, Sb_2_Se_3_, and Sb_2_(S,Se)_3_ solar cells. However, the Li‐TFSI used in Spiro‐OMeTAD is prone to deliquescence in the air, and the toxicity of 4‐tert‐butylpyridine and chlorobenzene poses a potential safety hazard.^[^ ^20^, ^21^ ^]^ Although many inorganic films have been used as the hole transport layer to increase the efficiency of Sb_2_Se_3_ solar cells, the efficiency is still far below the recording device that used Spiro‐OMeTAD as the hole transport layer. Therefore, identifying a more suitable hole transport layer or reaction etching at the back surface is crucial for the future development of Sb_2_Se_3_ solar cells.^[^ ^22^, ^23^ ^]^ To address these issues, this paper proposes using small atomic diameter lithium (Li) ions as dopants to dope the Sb_2_Se_3_ film, with lithium hydroxide (LiOH) acting as the dopant medium. Doping with Li ions can simultaneously solve the above three problems. Li‐ion doping can invert the carrier type of the grain boundary to decrease the deep defect density. With the diffusion of Li ions and the reaction at the back surface, the device's carrier transport and collection are increased.

In this study, we used small atomic diameter Li ions as dopants to dope the Sb_2_Se_3_ film, with LiOH acting as the dopant medium. We hypothesized that Li ions could easily enter the large gap between (Sb_4_Se_6_)_n_ ribbons, invert the carrier type of the grain boundary, and obtain a built‐in electric field from grain interiors (p‐type) to grain boundary (n‐type). During the LiOH and Sb_2_Se_3_ film reaction, Li ions could slightly enter the lattice of the Sb_2_Se_3_ film through Li ion diffusion. By varying the Li ions concentration, we could obtain Sb_2_Se_3_ films with a variable gradient band structure. The reaction between Sb_2_Se_3_ and LiOH solution increased the carrier collection and transport at the back contact surface. Furthermore, we extensively investigated the structure, crystallinity, orientation, and optical and electrical properties of Sb_2_Se_3_ film with a Li‐ion gradient. Finally, we achieved an efficiency of 7.57% for Sb_2_Se_3_ solar cells with a low deep‐level defect concentration.

## 2 Results and Discussion

**Figure 1:**
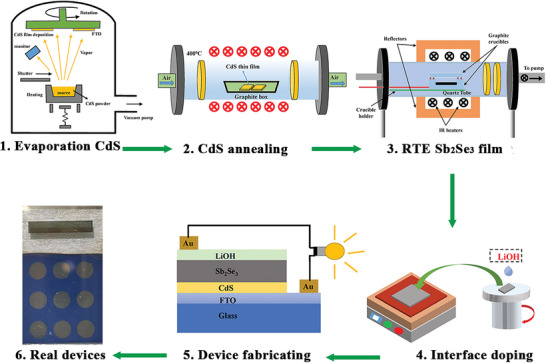
Illustration of the fabrication process for Sb_2_Se_3_ solar cells with Li gradient field by upper interface doping via solution method.

**Figure 2:**
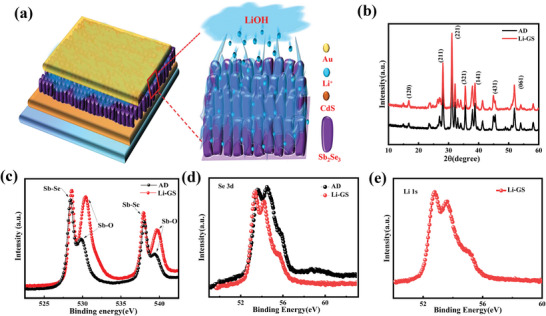
a) The schematic diagram of a Li‐doped Sb_2_Se_3_ film, b) XRD patterns of Sb_2_Se_3_ film with and without a Li gradient concentration, and c–e) XPS patterns of Sb_2_Se_3_ solar cells with and without a Li gradient concentration.

This study explores the formation of a Li gradient field through upper interface doping via a solution method. The detailed experiment process is shown in **Figure** **1**. The schematic diagram of the Li gradient field is illustrated in **Figure** **2**a. As shown in the figure, Li ions can easily enter the large gap between (Sb_4_Se_6_)_n_ ribbons and subsequently enter the lattice due to the corrosivity of the LiOH solution. During the diffusion process, the Li‐ion concentration decreases as the diffusion depth increases, ultimately forming a gradient of Li ions within the Sb_2_Se_3_ film and resulting in the formation of the Li gradient field. Furthermore, the *J‐V* curves of the device with different concentrations of LiOH solution are shown in Figure S1 (Supporting Information). The figure shows that when the concentration of LiOH solution was raised to 0.01 m, the efficiency of the device increased significantly. However, with a sustained increase in concentration, especially above 0.1 m, the efficiency of Sb_2_Se_3_ solar cells decreased significantly. We believe that this phenomenon is caused by the corrosion damage of high‐concentration LiOH solution on the Sb_2_Se_3_ film. To validate our hypothesis and understand the impact of the Li gradient field on the Sb_2_Se_3_ film, detailed characterization was performed on the Sb_2_Se_3_ film and device.

To investigate the influence of Li‐ion gradient on Sb_2_Se_3_ film, the researchers conducted a detailed analysis of the structure, crystallinity, electrical, and optical properties of the film with and without LiOH solution (AD means the Sb_2_Se_3_ film without LiOH solution, Li‐GS means the Sb_2_Se_3_ film with 0.01 m LiOH solution). Figure 2b displays the X‐ray diffraction (XRD) patterns of both Sb_2_Se_3_ films. It is evident from the figure that all the peaks of both films correspond to orthorhombic Sb_2_Se_3_ (JCPDS#15–0861) with a preferred orientation along the [221] and [211] directions.^[24]^ Moreover, the intensity ratio between [221] and [211] peaks of the film with Li‐ion gradient is higher than that of the film without Li‐ion gradient (Figure S2, Supporting Information). This phenomenon indicates that the carrier transport of Sb_2_Se_3_ film is higher in the sample with a Li‐ion gradient.^[25]^ In order to further study the effect of Li‐ion alkaline solution on Sb_2_Se_3_ film, the upper interface between Sb_2_Se_3_ film and Au electrode has been treated with different concentrations of LiOH solution. The detailed results are presented in Figure S3 (Supporting Information). As shown in the figure, the Sb_2_Se_3_ film was severely damaged as the concentration of LiOH solution increased, and at high concentration levels, the Sb_2_Se_3_ film was almost entirely corroded away. The XRD results of these six Sb_2_Se_3_ samples are displayed in Figure S4 (Supporting Information). The XRD results indicate that low concentrations of an alkaline solution containing Li‐ion do not affect the structure of Sb_2_Se_3_ film, whereas high concentrations of alkaline solution can severely damage the structure of Sb_2_Se_3_ film.

**Figure 3:**
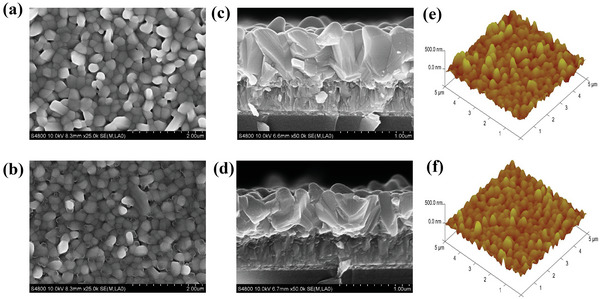
a) Surface SEM images, c) cross‐sectional SEM images, and e) AFM images of Sb_2_Se_3_ film without Li gradient concentration, b) the Surface SEM images, d) cross‐sectional SEM images, and f) AFM images of Sb_2_Se_3_ film with Li gradient concentration.

**Figure 4:**
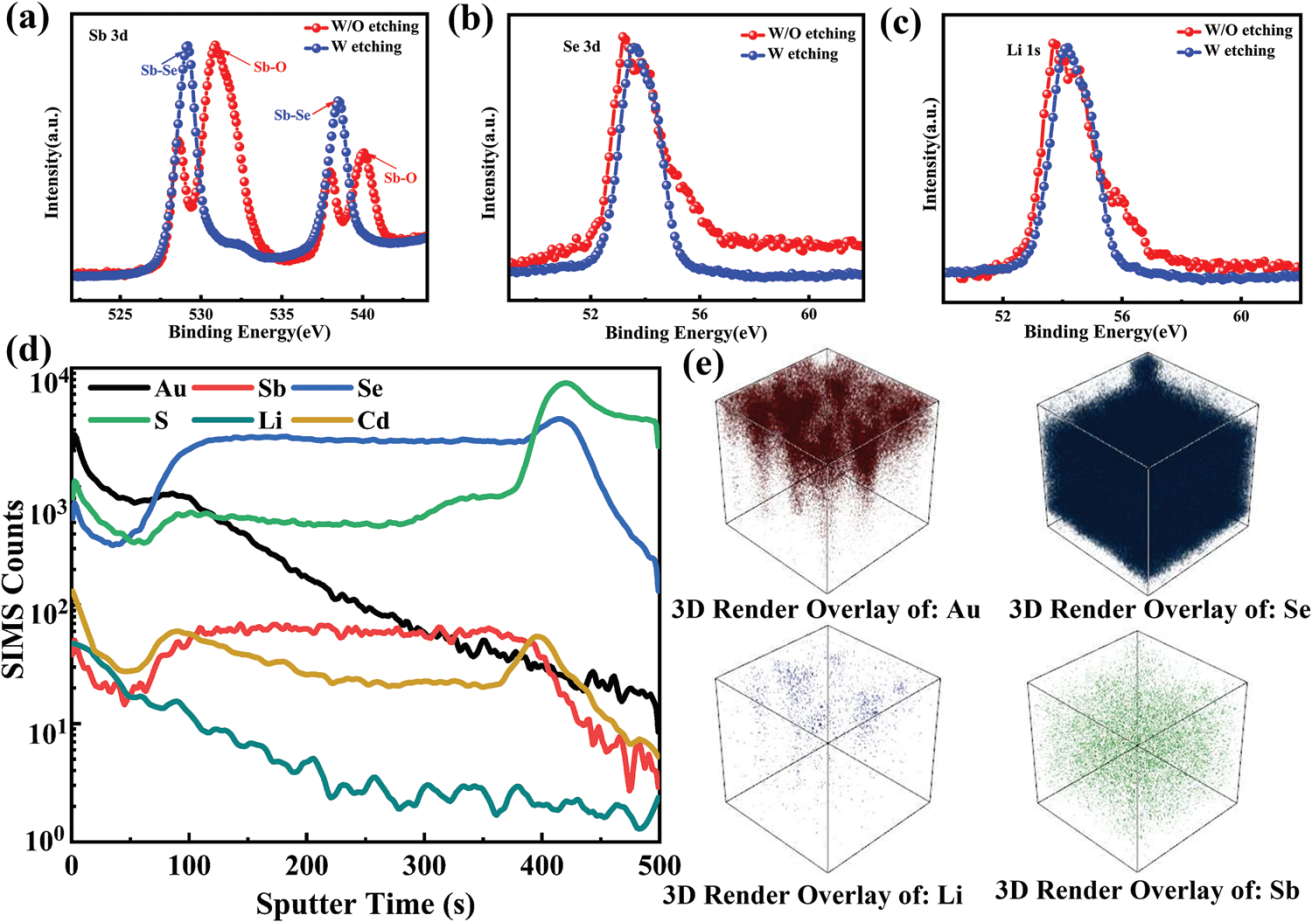
a–c) XPS patterns of the Sb_2_Se_3_ film with and without etching, d) TOF−SMIS patterns of the Sb_2_Se_3_ film with Li gradient field, e) 3D‐rendered overlays of selected elements of Sb_2_Se_3_ film with Li gradient field.

Figure 2c,e display the X‐ray photoelectron spectroscopy (XPS) results for Sb_2_Se_3_ films with and without a Li gradient. The Sb 3d spectrum in Figure 2c reveals that the binding energies of Sb(III) are situated at 538 and 528.7 eV, whereas the Sb 3d_3/2_ and Sb 3d_5/2_ peaks are located at 538.6 and 531.1 eV, respectively, which are in agreement with literature reports.^[26]^ However, the additional Sb─O bonds observed in Figure 2c are due to slight corrosion on the upper interface of the Sb_2_Se_3_ film after adding LiOH solution. A prior study has demonstrated that an appropriate thickness of the Sb─O layer can reduce carrier recombination and enhance carrier transport in the device.^[27]^ However, if the thickness of Sb_2_O_3_ is sustained increasing, the thick Sb_2_O_3_ may increase the current leakage, deep defect density and decrease the carrier collection at the back contact interface.^[^ ^28^, ^29^ ^]^ The Se 3d peak is presented in Figure 2d, where the Se 3d_3/2_ and Se 3d_5/2_ peaks are located at 54.1 and 53.3 eV, respectively. Figure 2e shows the Li 1s spectrum of the Sb_2_Se_3_ film with a Li‐ion gradient, where the peaks at 53.5 and 54.2 eV correspond to Li 1s, respectively.^[28]^ The XPS data demonstrate that even a low concentration of LiOH solution can result in slight corrosion on the surface of the Sb_2_Se_3_ film, and Li‐ion elements can enter into the Sb_2_Se_3_ film.

To examine the effect of LiOH solution on the morphology of Sb_2_Se_3_ film, scanning electron microscopy (SEM) was performed on Sb_2_Se_3_ films with and without 0.01 m LiOH solution. As illustrated in **Figure** **3**a, the surface morphology of the Sb_2_Se_3_ film without LiOH displayed a disorderly arrangement with numerous pin‐holes on the surface. However, the Sb_2_Se_3_ film with LiOH solution showed a flat and orderly surface with fewer pin‐holes, indicating that the LiOH solution had a corrosive effect. Figure 3c,d present cross‐sectional SEM images of Sb_2_Se_3_ films with and without LiOH solution, revealing that the Sb_2_Se_3_ film exhibited better crystallinity with larger grain sizes when LiOH solution was added. Atomic force microscopy (AFM) images of the Sb_2_Se_3_ films (Figure 3e,f) indicated that the surface roughness decreased notably when the LiOH solution was added, consistent with the SEM results. A smoother surface is generally preferred for achieving high‐quality interfaces between adjacent layers. A smooth surface reduces the recombination of light‐induced excitations at the absorber‐metal electrode interface, thereby improving cell performance.^[30]^

To further confirm the presence of Li ions and the formation of a concentration gradient, XPS and time‐of‐flight secondary ion mass spectrometry (TOF‐SIMS) were conducted on Sb_2_Se_3_ films treated with alkaline solutions containing Li ions. **Figure** **4**a–c illustrates the XPS results of Sb_2_Se_3_ films with and without etching to a depth of 100 nm. The results demonstrate that the Sb─O bonds disappeared when the etching depth reached 100 nm, indicating that the Sb─O layer only exists on the surface of the Sb_2_Se_3_ film.^[31]^ Additionally, the Li‐ion signal was detected even when the etching depth had reached 100 nm. The TOF‐SIMS results in Figure 4d indicate that the Li‐ion signal can be detected throughout the entire Sb_2_Se_3_ thin film. Unlike the accumulation of potassium and aluminium ions in the midsection of the Sb_2_Se_3_ film, the concentration of Li ions gradually decreases from the surface to the bottom of the Sb_2_Se_3_ film.^[32]^ Figure 4e presents a 3D render overlay of elements Sb, Se, Li, and Au, clearly showing the position of the elements. These results more intuitively demonstrate the Li‐ion concentration gradient inside the Sb_2_Se_3_ film. The XPS and TOF‐SIMS results confirm our hypothesis regarding the concentration gradient of Li ions. The Li gradient field can be generated using this method.

**Figure 5:**
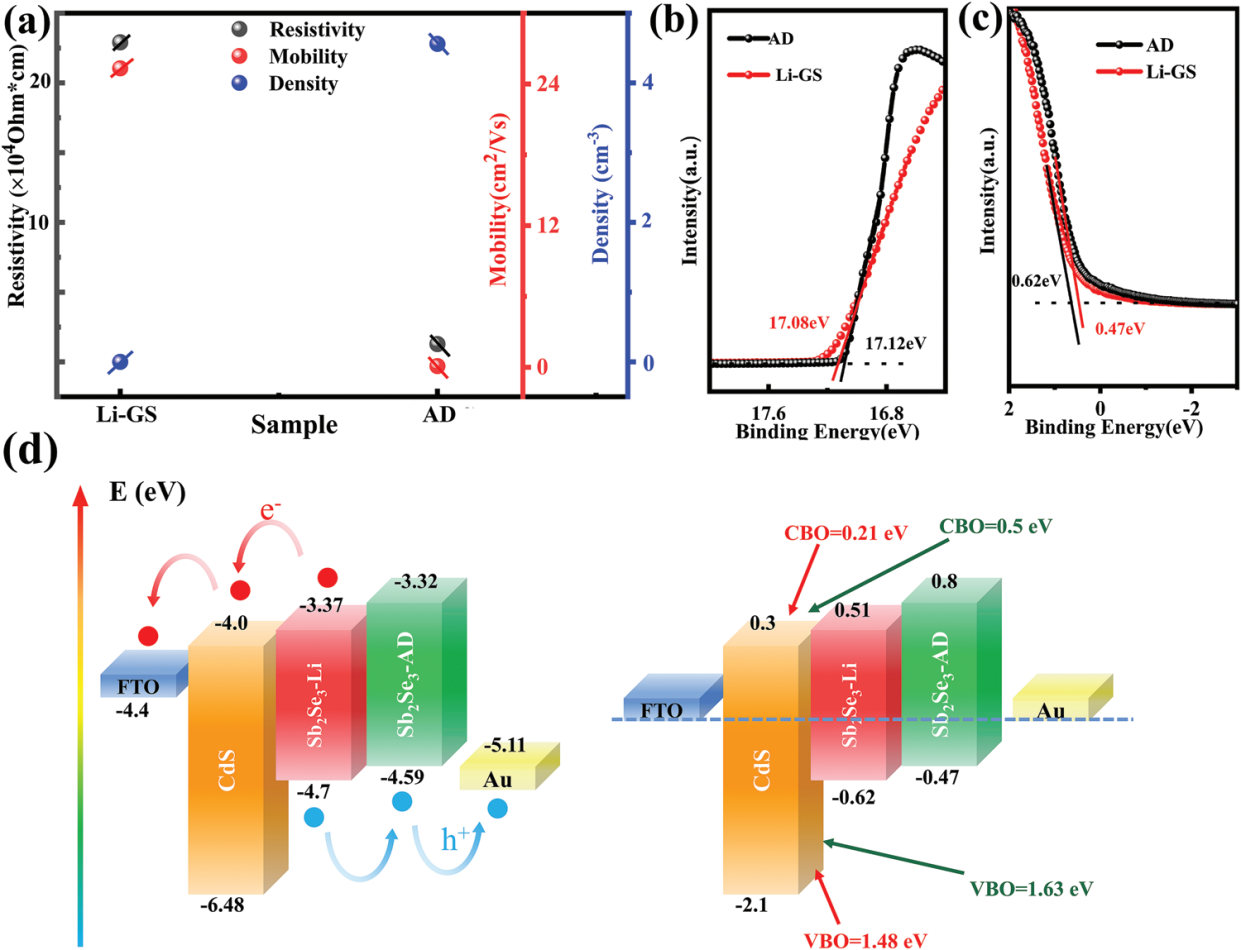
a) Electrical properties of Sb_2_Se_3_ films with and without Li gradient field, b,c) UPS cut‐off edge and valence band spectra of Sb_2_Se_3_ films with and without Li gradient field, d) Band structures of Sb_2_Se_3_ solar cells with and without Li gradient field.

**Figure 6:**
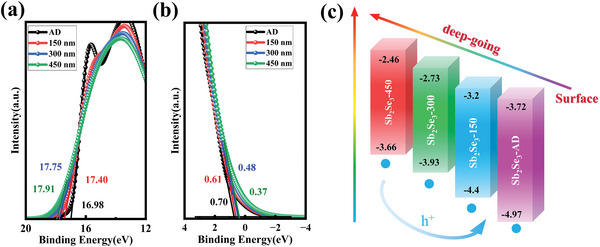
In‐depth UPS analysis of Sb_2_Se_3_ film with Li gradient: a) UPS spectra of the work function edge, b) UPS spectra of the valence‐band edge, and c) Gradient energy levels at the gradient of the Sb_2_Se_3_ film with Li gradient.

**Figure** **5**a illustrates the electrical performance of Sb_2_Se_3_ films with and without a Li gradient field. As shown in the figure, the resistivity and mobility of the Sb_2_Se_3_ film increased with the addition of Li gradient, whereas the hole carrier concentration decreased. Moreover, during the Hall test, the carrier type of the Sb_2_Se_3_ film with Li ions was always n‐type, which resulted mainly from the inversion of the carrier type at the grain boundary. In addition, the n‐type Sb_2_Se_3_ film has already been reported.^[33]^ To further verify this phenomenon and investigate the effect of the Li gradient on band energy alignment, the ultraviolet photoelectron spectroscopy (UPS) spectra and energy alignment of the films with and without a Li gradient field are presented in Figure 5b,c. Fitting the cut‐off binding energy and long tails of the UPS spectra allowed for the determination of the Fermi level (*E*_F_) and valence band maximum (VBM, *E*_v_) of the Sb_2_Se_3_ film. The conduction band minimum (CBM, *E*_c_) was determined from the energy gap and VBM position. The energy levels of all samples were calculated from the UPS spectra and bandgap. The UPS results revealed that the distance between the *E*_F_ and VBM was calculated to be 0.62 and 0.47 eV, respectively. With the addition of a Li gradient, the Fermi level moved toward the conduction band compared to its position in the film without a Li gradient. Additionally, the results demonstrated that the CBO between the Sb_2_Se_3_ film and CdS film was 0.5 eV, whereas with the addition of a Li gradient, the CBO between the Sb_2_Se_3_ and CdS films was 0.21 eV, which falls within the range of moderate spike‐like CBO values (0–0.4 eV).^[34]^ The efficiency of the device can be decreased since a big CBO raises the barrier for collecting photogenerated electrons. **Figure** **6**a,b present the UPS cut‐off spectra of Sb_2_Se_3_ films with different etching depths (0, 150, 300, and 450 nm). In the band structure calculation process, it is challenging to estimate the band gap of the gradient Sb_2_Se_3_ film. Therefore, we hypothesized that the band gap of the Sb_2_Se_3_ film with different etching depths is consistent with the theoretical band gap. The *E*_C_ for the sample with etching depths of 0, 150, 300, and 450 nm are −4.97, −4.4, −3.93, and −3.66 eV, respectively. These results support the steady enhancement of CBM structure in the Sb_2_Se_3_ films. Figure 6c shows the energy‐level alignment between the CdS film and Sb_2_Se_3_ film with a Li gradient. Due to the gradient CBM structure, a large barrier is transformed into many small barriers, making it easier for carriers to cross over them.^[^ ^35^, ^36^ ^]^ These relevant XPS and UPS results confirm the beneficial role of the gradient Sb_2_Se_3_ film in achieving high‐quality heterojunctions and efficient carrier transport.

**Figure 7:**
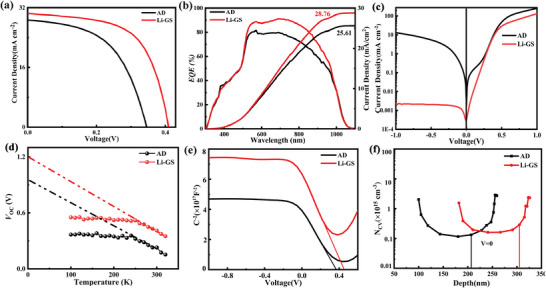
a) *J−V* curves, b) *EQE* curves, c) dark *J−V* curves, d) temperature‐dependent open‐circuit voltage measurements, e) *C−V* curves, and f) *N*_C–V_ curves of Sb_2_Se_3_ solar cells with and without a Li gradient field.

**Figure** **7**a displays the *J–V* curves of Sb_2_Se_3_ solar cells with and without a Li gradient field. As shown in the figure, the addition of a Li gradient field has increased all of the device parameters, especially the *V*_oc_. The Sb_2_Se_3_ solar cells with LiOH solution achieved an efficiency of 7.57%, with a *V*_oc_, current density (*J*_sc_), and fill factor (FF) of 0.41 V, 30.5 mA cm^−^ ^2^ and 60.51%, respectively. While the Sb_2_Se_3_ solar cells without LiOH solution obtained an efficiency of 5.2%, with a *V*_oc_, *J*_sc_, and FF of 0.34 V, 28.66 mA cm^−^ ^2^ and 52.75%, respectively. Figure 7b displays the external quantum efficiency (*EQE*) results of both devices. As seen in the figure, the device with a Li gradient field exhibited a high optical response in the long wavelength, indicating a reduction in the recombination rate and high carrier collection. Additionally, biased *EQE* measurements (defined as *EQE* (−0.5 V)/*EQE*(0 V)) were conducted on both devices. The results showed that the observed *EQE* ratio of Sb_2_Se_3_ solar cells with a Li gradient field was approximately unity during the entire spectrum. However, the observed *EQE* ratio of Sb_2_Se_3_ solar cells without a Li gradient field was strongly dependent on the bias, especially in the long‐wavelength region. The biased *EQE* results showed that the carrier collection of the device was optimized with the addition of a Li gradient field (Figure S8, Supporting Information). Figure 7c shows the dark *IV* results of both devices. The results indicated that with the addition of a Li gradient field, the diode ideal factor (A) and reverse saturation current density (J_0_) decreased significantly. The low ideal factor and reverse saturation current density can decrease carrier recombination and promote charge transport. Temperature‐dependent open circuit voltage measurements were conducted from 150 to 330K (shown in Figure 7d). The obtained value of activation energy for Sb_2_Se_3_ solar cells with a Li gradient field is 1.2 eV, which is very close to the band gap of Sb_2_Se_3_ films (1.26 eV, as shown in the Figures S9 and S10, Supporting Information), while the Sb_2_Se_3_ solar cells without a Li gradient field is 0.97 eV. This result indicates that the interfacial recombination of Sb_2_Se_3_ solar cells with a Li gradient field is much lower than that of Sb_2_Se_3_ solar cells without a Li gradient field. The temperature‐dependent open circuit voltage results indicate that the upper interface treatment of LiOH can diffuse into the entire Sb_2_Se_3_ film and form a Li gradient field. The capacitance–voltage (C‐V) and depletion region width (W_d_) results are shown in Figure 7e,f, and the built‐in voltages (*V*_bi_) of the device with and without treatment were 0.415 and 0.38 V, respectively. The improvement in *V*_bi_ can decrease the accumulation of photogenerated charge at the interfacial region, leading to an enhancement in the device *V*_OC_, as reported in previous studies.^[^ ^37^, ^38^ ^]^ The depletion region width, which is used to analyze orientation‐related defects, was obtained from the *C–V* graph. For the Sb_2_Se_3_ film without and with Li gradient field, the depletion region widths were calculated to be 217 and 304 nm, respectively. A larger W_d_ value in the device with a Li gradient field could result in a greater collection of photogenerated carriers and contribute to a larger *J*_sc_, as observed in the results of *EQE*.^[39]^

**Figure 8:**
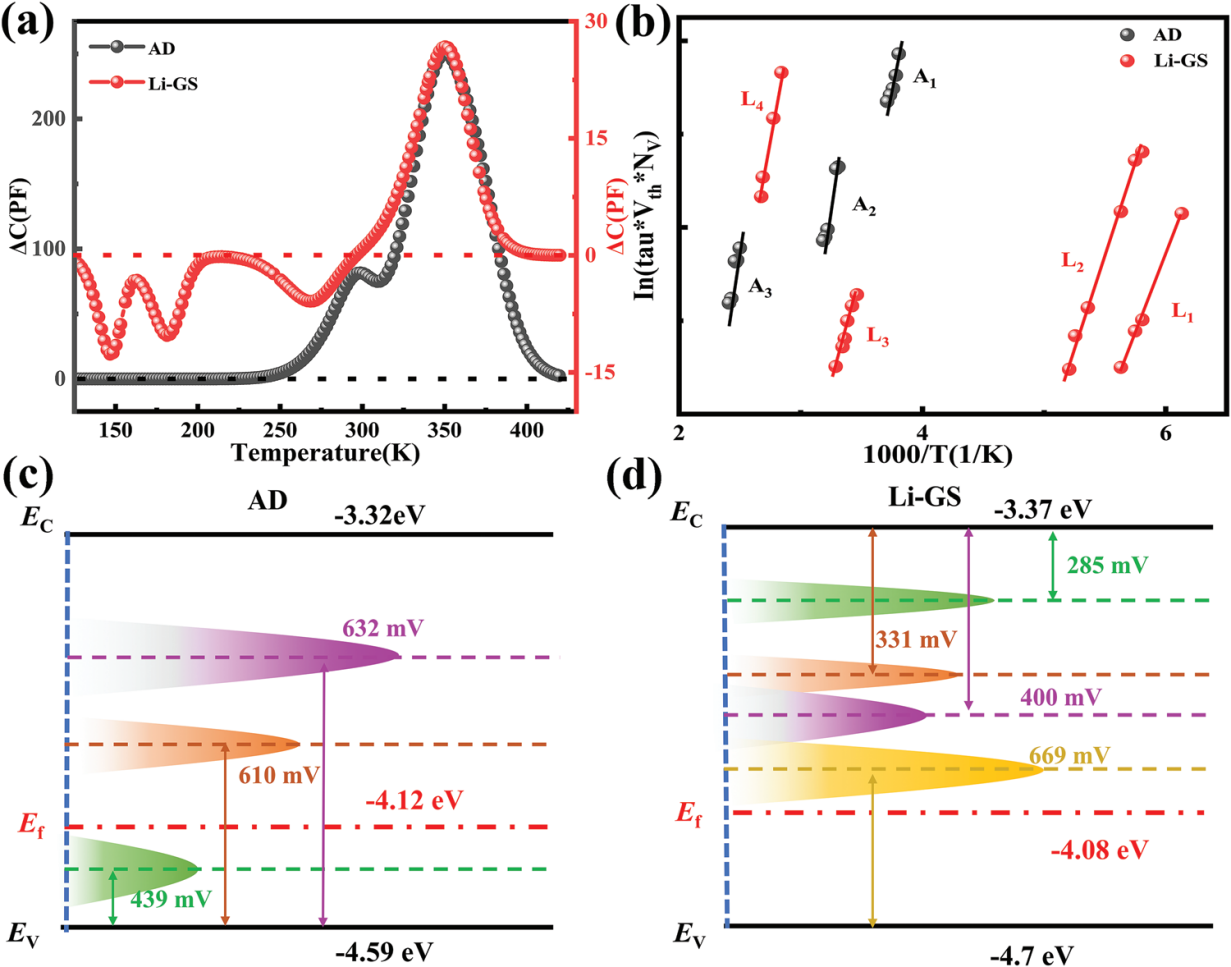
a) DLTS signals of Li‐GS and AD Sb_2_Se_3_ devices. b) Arrhenius plots obtained from DLTS signals. c) Energy states and defect levels of the AD device. d) Energy states and defect levels of the Li‐GS solar cells.

To further examine the impact of the Li gradient field on the profound defects of Sb_2_Se_3_ film, non‐radiative recombination centers, specifically electrically active defects in Sb_2_Se_3_ solar cells with and without Li gradient field, were characterized using deep‐level transient spectroscopy (DLTS). DLTS is a technique that tracks the changes in the charge state of deep defect centers at various temperatures using the transient capacitance of the p‐n junction.^[40]^ In this study, traps in the samples were found using the minority carrier injection DLTS (inj‐DLTS). Both electrons and holes can be injected into the depletion zone when a forward pulse voltage is used.

Three negative peaks (Li‐GS‐E1 at 150 K, Li‐GS‐E2 at 180 K, and Li‐GS‐E3 at 270 K) corresponding to minority‐carrier (electron) traps in the p‐type Sb_2_Se_3_ layer were seen in the DLTS spectra for the Sb_2_Se_3_ solar cell with a Li gradient field, as shown in **Figure** **8**a. The trap levels for Li‐GS‐E1, Li‐GS‐E2, and Li‐GS‐E3 were 285, 331, and 400 mV below the conduction band, respectively. In contrast, a positive peak was already observed at 370K in the DLTS spectrum. Conversely, in the DLTS spectrum of Sb_2_Se_3_ solar cells without a Li gradient field, no negative peaks were observed, and three positive peaks (AD‐H1 at ≈210 K, AD‐H2 at ≈300 K, and AD‐H3 at ≈370 K) corresponding to minority‐carrier (hole) traps in the p‐type Sb_2_Se_3_ layer were observed. As shown in Figure 8b, we calculated the trap concentration and the associated capture cross‐section for each trap state using Arrhenius plots of thermal emission rates as a function of reciprocal temperature. The defect information obtained is summarized in **Table** **1**.

**Table 1:** The detailed DLTS parameters of Sb_2_Se_3_ solar cell with and without 0.01 m LiOH solution.

Sample	Trap	ET[eV]	*σ* [cm^2^]	*N*_T_[cm^−3^]	*τ* [ns]
As‐deposited	A1	*E*_V_ + 0.439	2.06 × 10^−17^	4.03 × 10^14^	1.2 × 10^3^
	A2	*E*_V_ + 0.610	3.56 × 10^−15^	1.71 × 10^15^	1.64 × 10^1^
	A3	*E*_V_ + 0.632	5.16 × 10^−17^	8.53 × 10^15^	2.27 × 10^2^
Li gradient	L1	*E*_C_‐0.285	2.64 × 10^−16^	4.05 × 10^14^	9.35 × 10^2^
	L2	*E*_C_‐0.331	9.78 × 10^−16^	3.33 × 10^14^	3.07 × 10^2^
	L3	*E*_C_‐0.400	9.21 × 10^−18^	2.92 × 10^14^	3.71 × 10^3^
	L4	*E*_V_ + 0.669	3.35 × 10^−18^	8.67 × 10^14^	3.44 × 10^3^

Figure 8c,d illustrate the energy states and defect levels of the AD device and the Li‐GS solar cells. The width of each peaks corresponding to the capture cross‐section and the length corresponding to the concentration of the defects. In our rapid thermal evaporation (RTE) method, Se vapor is always in excess due to its higher vapor pressure than Sb and Sb_2_Se_3_, resulting in slightly Se‐rich Sb_2_Se_3_ films. The previous study reported that under Se‐rich conditions, the dominant acceptor defects are antimony vacancy (V_Sb_) and selenium antisite (Se_Sb_) defects.^[41]^ Therefore, we suspect that AD‐H1 is due to the V_Sb_ defect, while AD‐H2 and H3 are tentatively attributed to Se_Sb1_ and Se_Sb2_, respectively. Which is consistent to the previous theoretical work.^[^ ^42^, ^43^ ^]^ As the location of Li‐GS‐H1 is close to the defect of AD‐H3, we suspect that the Li‐GS‐H1 defect is due to Se_Sb2._ However, in the DLTS results with the addition of LiOH solution, three electrical traps appeared. We speculate that this phenomenon is due to two reasons. First, previous research has reported that the distribution of defects in trichalcogenides is dominated by antisite defects due to the similar sizes of the constituent atoms. Second, simulations have shown that Se_Sb_ and antimony antisite (Sb_Se_) are acceptors and donor defects, respectively.^[42]^ We suspect that the Li‐GS‐E3 defect is due to the Se_Sb_ defect. Second, as the location of Li‐GS‐E1 and Li‐GS‐E2 is very close to the CBM, we believe that these two defects can be attributed to Li itself. Our hypothesis is that Li ions first accumulate on the surface of the Sb_2_Se_3_ film and then diffuse easily into the (Sb_4_Se_6_)_n_ chains and lattice of the Sb_2_Se_3_ film due to the corrosive nature of LiOH solution and the large distance and small compactness at GBs. Microscale Li ions diffuse easily along the GBs and enter the gaps of the chains, resulting in n‐type Li_i_ doping. This inversion creates a local electric field between GBs and GIs, facilitating spatial separation of photogenerated electrons and holes, thereby restraining carrier recombination and enhancing carrier collection, especially for carriers generated by red and infrared photons, resulting in improved efficiency. Additionally, it is worth noting that the defect density of the Sb_2_Se_3_ film with added LiOH solution decreased by order of magnitude. This reduction in defect density should decrease carrier recombination and increase carrier transport in the device.

## 3 Conclusion

In summary, the LiOH solution was used to modify the upper interface of the device. Due to the sensitivity of the Sb_2_Se_3_ film to the alkaline solution, Li ions not only formed a back contact layer at the upper interface but also entered into the lattice of the Sb_2_Se_3_ film to enhance its crystallinity, optical, and electrical properties. The UPS results also indicated that with the addition of LiOH solution, a gradient band structure was formed inside the Sb_2_Se_3_ film, which should improve the carrier collection and transport of the device. The DLTS results also showed a reduction in defect density and grain boundary inversion which should suppress carrier recombination and enhance carrier collection, resulting in an improvement in PCE. Finally, an efficiency of 7.57% was achieved for Sb_2_Se_3_ solar cells. This is the highest efficiency reported for Sb_2_Se_3_/CdS solar cells based on rapid thermal evaporation. We believe that this work paves the way for further improvements in the efficiency of Sb_2_Se_3_ solar cells.

## 4 Experimental Section

### The Fabrication of Films and Devices

In this paper, the device is fabricated based on the structure of FTO/CdS/Sb_2_Se_3_/Au. First, through single source evaporation (PTN104736, Mini SPECTROS, Kurt J. Lesker, USA), a 50 nm CdS film was evaporated onto the FTO substrate (with a sheet resistance of 6 sq^−1^ and transmittance 80%, Advanced Election technology Co., LTD China) using CdS powder (99.999%, China New Metal Materials Technology Co., Ltd, China). During the evaporation process, the substrate temperature was room temperature, the working pressure was 1.0×10^−6^ torr, the evaporation speed was 0.65 s^−1^, and the evaporation power was 35 W. The CdS film was then annealed in a tube furnace for one minute at 500 °C. Second, based on the RTE technique, Sb_2_Se_3_ films with a thickness of ≈500 nm were created in a tube furnace that was maintained at a pressure of 5 m Torr by a mechanical pump. The pre‐heating temperature was 310 °C for 20 min, and the depositing temperature was raised to 570 °C for 40 s. Third, the LiOH solution's concentration ranged from 0 to 0.1 m, with water serving as the solvent. Additionally, the Sb_2_Se_3_ films were heated at 100 °C while spin‐coating them with various LiOH concentrations for comparison. At last, Gold electrodes (0.0225 cm^2^) with a thickness of 80 nm were prepared by evaporation.

### Characterization of Films and Solar Cells

In this paper, the structure and crystallinities of all the Sb_2_Se_3_ film at various LiOH concentrations were examined by the XRD based on the smart Apex II Duo system. The SEM was used to analyze the surface and cross‐sections morphologies of all the samples. The AFM was used to measure the surface roughness and a UV–visible spectrophotometer (Shimadzu UV‐2600) was used to measure optical performance of all the Sb_2_Se_3_ films. UPS was carried out using a monochromatic He I light source (21.2 eV) and a VG Scienta R4000 analyser, while XPS was carried out using a Kratos Axis Ultra DLD system. For testing the electrical properties, Sb_2_Se_3_ films with different concentrations of LiOH were deposited directly on the glass substrate and measured using a Hall measurement system with van der Pauw configuration at room temperature and a magnetic flux density between 3000–5000G (QT‐50, Quatek, Germany). The metal In was used as the electrode for the hall test. Deep analysis of the elements was performed with a TOF‐SMIS measurement system (IONTOF GmbH, Münster, Germany). The EQE of the solar cells was detected using a photovoltaic characteriszation system (PV Measurements QEXL), and the efficiency of the solar cells was measured using an *I–V* tester under AM 1.5G spectral irradiance with a radiance of 1000 W m^−2^. Using a Keysight Precision LCR metre (Keysight E4980AL) in complete darkness, at 25 °C, and at 10 kHz, the *C–V* was measured. A range of ‐1–0.5 V was used as the DC bias voltage. The DLTS spectra were obtained using the Phystech FT‐1230 HERA DLTS system, and temperature scans were made between 120 and 400 K, with 2K intervals.

## Conflict of Interest

The authors declare no conflict of interest.

## Supporting information

Supporting Information

## Data Availability

The data that support the findings of this study are available on request from the corresponding author. The data are not publicly available due to privacy or ethical restrictions.
