# Supplementary material for: Enhancement in the Efficiency of Sb2Se3 Solar Cells by Triple Function of Lithium Hydroxide Modified at the Back Contact Interface
Source: Adv Sci (Weinh). 2023 Sep 10;10(31):2304246. doi: 10.1002/advs.202304246 (PMC10625132; doi:10.1002/advs.202304246)
Supplement: Supplementary file 1 — Supporting Information [file ADVS-10-2304246-s001.pdf]

ADVANCED SCIENCE
Open Access

Supporting Information

for *Adv. Sci.*, DOI 10.1002/advs.202304246

Enhancement in the Efficiency of Sb_2Se_3 Solar Cells by Triple Function of Lithium Hydroxide Modified at the Back Contact Interface

Huafei Guo, Shan Huang, Honcheng Zhu, Tingyu Zhang, Kangjun Geng, Sai Jiang, Ding Gu, Jian Su, Xiaolong Lu, Han Zhang, Shuai Zhang, Jianhua Qiu*, Ningyi Yuan* and Jianning Ding*

Supporting Information

Enhancement in the Efficiency of Sb_2Se_3 Solar Cells by Triple Function of Lithium Hydroxide Modified at Back Contact Interface

Huafei Guo, Shan Huang, Honcheng Zhu, Tingyu Zhang, Kangjun Geng, Sai Jiang, Ding Gu, Jian Su, Xiaolong Lu, Han Zhang, Shuai Zhang, Jianhua Qiu*, Ningyi Yuan*, and Jianning Ding*

School of Microelectronics and Control Engineering, Jiangsu Collaborative Innovation Center for Photovoltaic Science and Engineering, Jiangsu Province Cultivation base for State Key Laboratory of Photovoltaic Science and Technology, Changzhou University, Changzhou 213164, China.

E-mail: guohuafei@cczu.edu.cn; jhqiu@cczu.edu.cn; nyyuan@cczu.edu.cn;

Keywords: Sb_2Se_3, Li gradient field, gradient band structure, deep-level defect, carrier collection

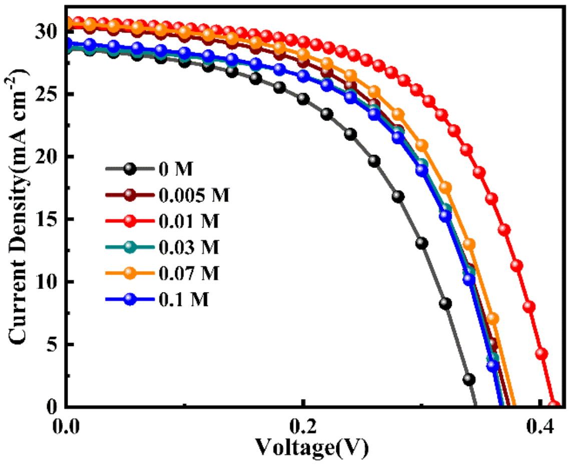

Fig S1 The *J*-*V* curves of Sb_2Se_3 solar cells with various concentration of LiOH solution

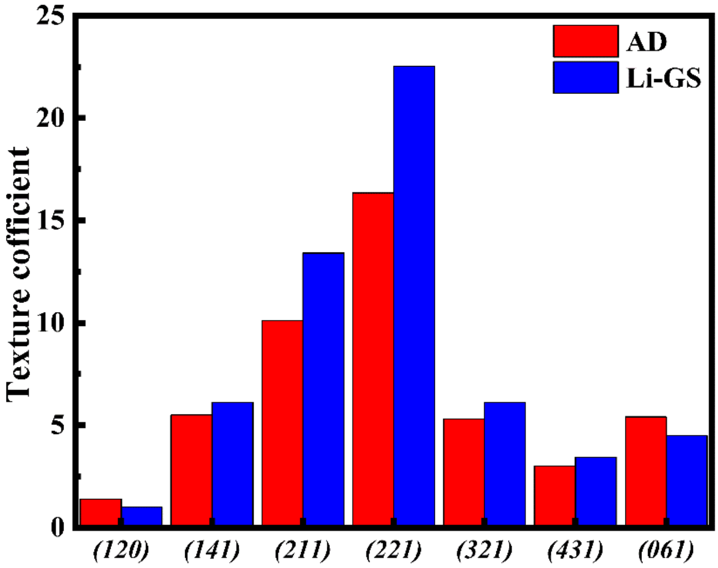

Fig S2 The Texture coefficient of Sb_2Se_3 thin film with and without LiOH solution

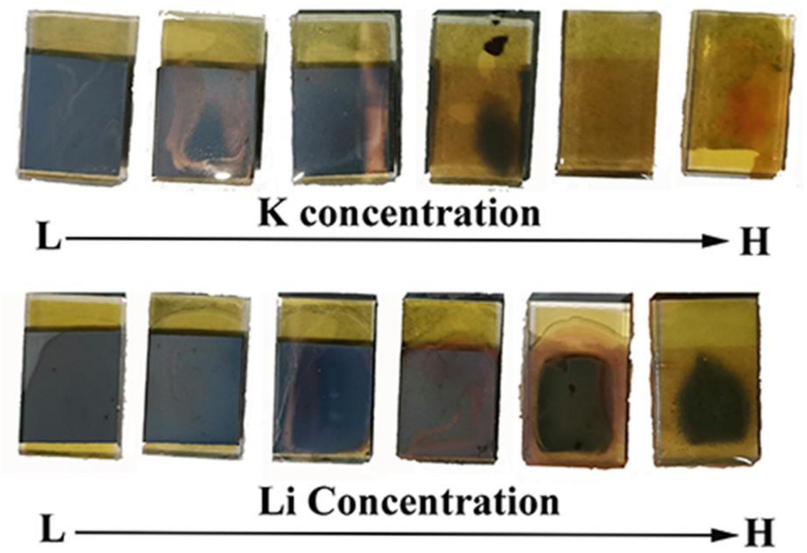

Fig S3 The photograph of Sb_2Se_3 film with various concentration of LiOH solution

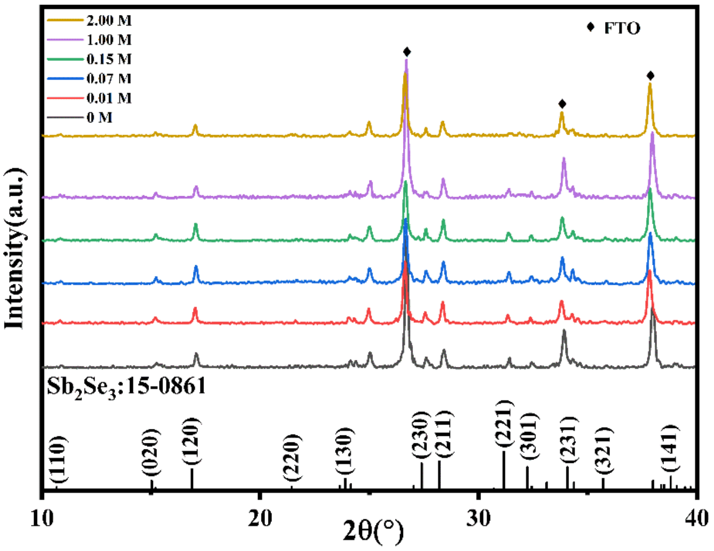

Fig S4 The XRD patterns of Sb_2Se_3 film with various concentration of LiOH solution

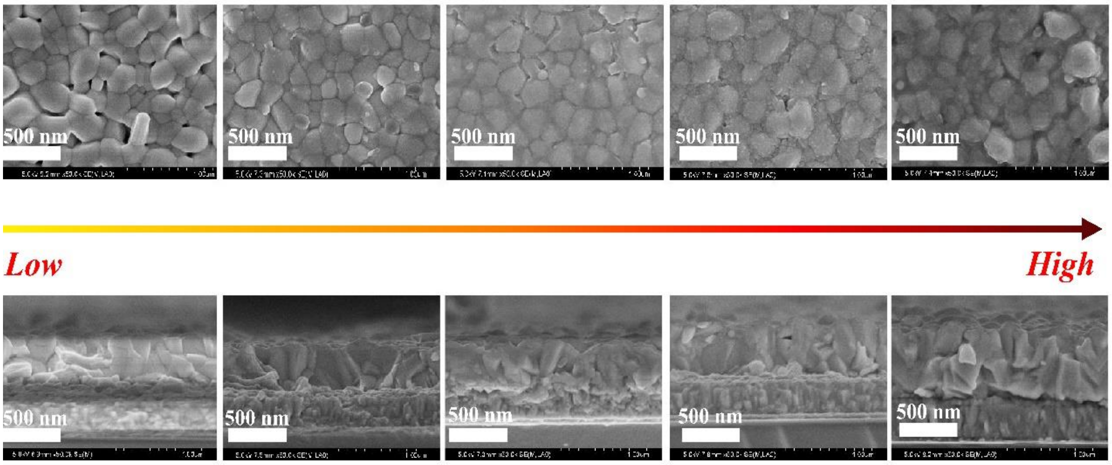

FigS5 The surface and cross-sectional SEM images of Sb_2Se_3 solar cells with various concentration of LiOH solution

It can be seen from the figure, with the increasing concentration of LiOH solution, the residue on the surface of Sb_2Se_3 film become more and more obviously, the residue is mainly resulted from the reaction between Sb_2Se_3 and LiOH. In addition, we suspect the chemical reaction of LiOH is: $6LiOH+Sb_2Se_3 \rightarrow 3Li_2Se+Sb_2O_3+3H_2O$. Based on the SEM and IV(FigS1) result, the more residue on the surface will increase the contact resistance and hinder the carrier transport at the interface between Sb_2Se_3 and Au layer.

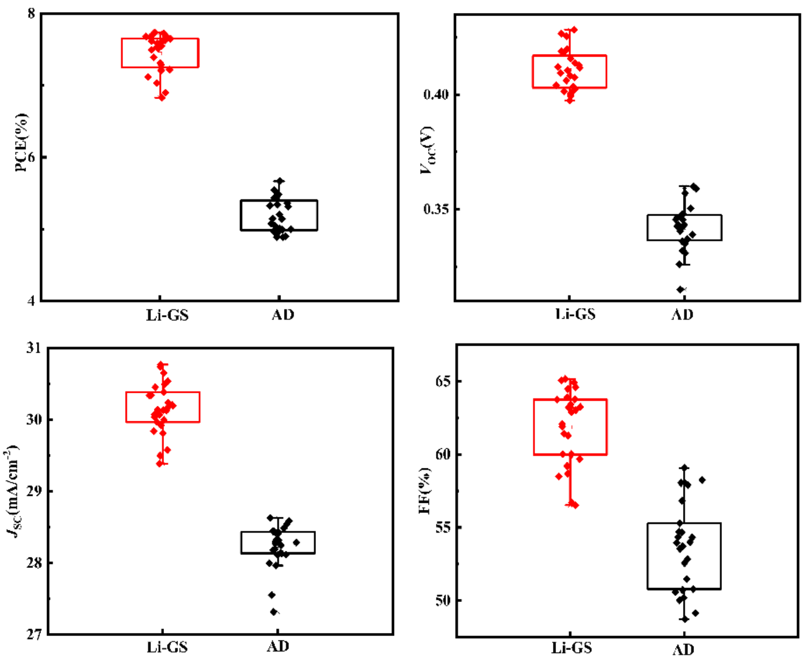

Fig S6 The statistical deviation of the device with and withou adding Li ion

Fig S6 show the statistical deviation of the best photoelectric parameter among 24 devices with and without LiOH solution, the result also indicates the superiority of device with LiOH solution over the device without LiOH solution

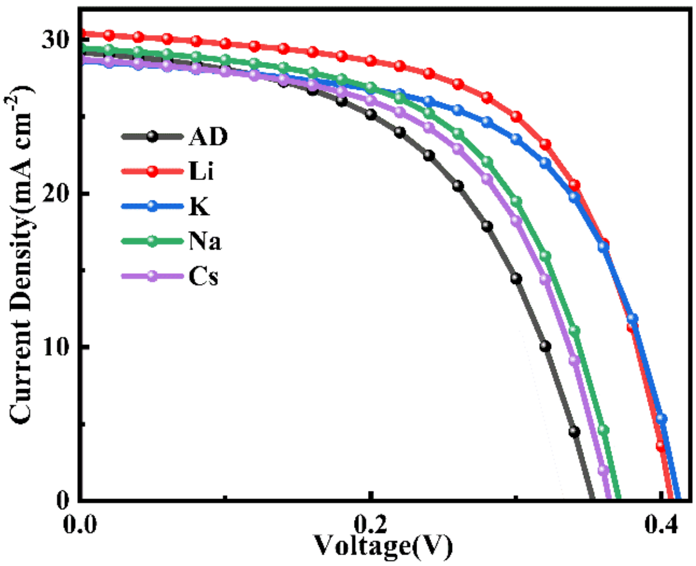

Fig S7 The *J*-*V* curves of Sb_2Se_3 solar cells with various alkaline elements

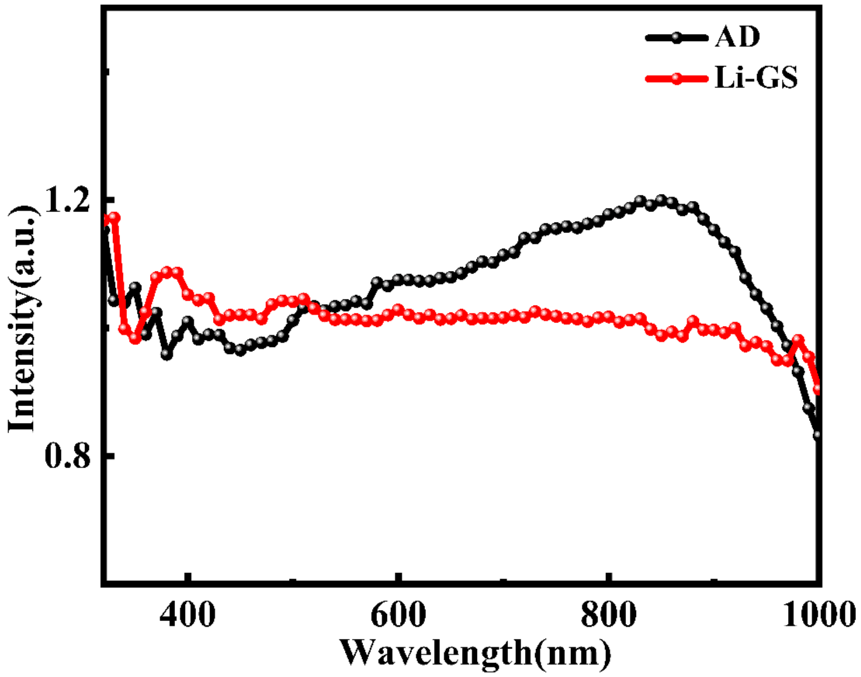

Fig S8 The ratio EQE(-0.5V)/EQE(0V) of the devices without and with LiOH solution. Applied bias is -0.5V.

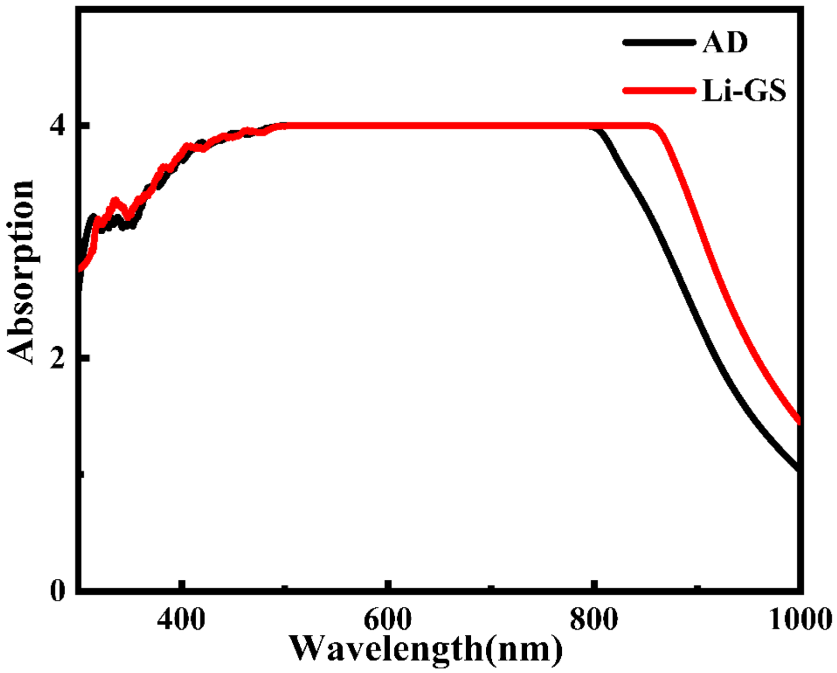

Fig S9 The absorption of Sb_2Se_3 film with and without LiOH solution

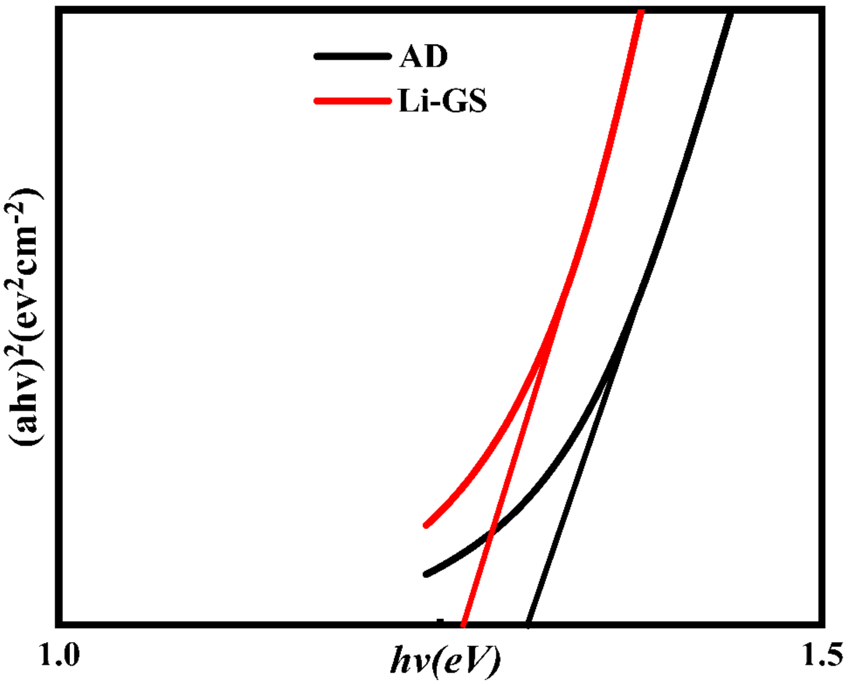

Fig S10 The bandgap of Sb_2Se_3 film with and without LiOH solution
